# Surveillance of West Nile virus infections in humans and animals in Europe, monthly report – data submitted up to 7 October 2026

**DOI:** 10.2903/j.efsa.2026.10410

**Published:** 2026-10-26

**Authors:** European Centre for Disease Prevention and Control (ECDC), European Food Safety Authority (EFSA)

**Keywords:** birds, equids, humans, outbreak, West Nile virus

## Abstract

As part of their One Health activities, the European Centre for Disease Prevention and Control (ECDC) and the European Food Safety Authority (EFSA) jointly conduct surveillance of West Nile virus (WNV) infections in humans, equids and birds and assess the epidemiological situation in Europe. This surveillance informs public health and veterinary authorities and supports timely prevention and control measures. The 2026 WNV season has affected a broader geographical area than any previous season, with the numbers of human cases and animal outbreaks exceeding those reported in previous years. Overall, there is no indication of increased disease severity in humans in Europe. Italy remains the most affected country, reporting the highest numbers of human cases and avian outbreaks. Greece and France reported their highest numbers of human WNV cases on record, with France also reporting a record number of equid outbreaks. The geographical distribution of reported WNV activity has expanded in northern Europe. Human cases and animal outbreaks were detected across multiple regions in Belgium and the Netherlands, with Belgium reporting its first human WNV infections. WNV activity has declined since its August–September peak, but further cases may still be reported. ECDC and EFSA continue to monitor the WNV epidemiological situation in Europe.

Suggested citation: European Centre for Disease Prevention and Control, European Food Safety Authority, 2026. Surveillance of West Nile virus infections in humans and animals in Europe, monthly report – data submitted up to 7 October 2026. Available online: https://efsa.onlinelibrary.wiley.com/doi/10.2903/j.efsa.2026.10410. The most recent full online report is available at: https://www.ecdc.europa.eu/en/infectious-disease-topics/west-nile-virus-infection/surveillance-and-disease-data/monthly-updates

Requestor: European Commission

Question number: EFSA-Q-2025-00338

ISSN: 1831-4732

## Epidemiological overview

### Findings from human surveillance

#### EU/EEA countries

In 2026, and as at 7 October, 13 countries in the EU/EEA have reported 1 715 locally acquired[1] human cases of West Nile virus (WNV) infection. The earliest and latest dates of symptom onset among human cases were 22 May in Italy and 30 September in Italy, respectively. Locally acquired cases have been reported by **Italy** (748 cases, of which 1 had an unknown place of infection), **Greece** (445 cases, of which 6 had an unknown place of infection), **France** (144 cases), **Spain** (139 cases), **Romania** (117 cases), **the Netherlands** (59 cases, of which 1 had an unknown place of infection), **Croatia** (16 cases), **Belgium** (15 cases), **Cyprus** (13 cases), **Austria** (8 cases), **Hungary** (5 cases), **Germany** (4 cases) and **Bulgaria** (2 cases). A total of 130 deaths have been reported by **Italy** (56 deaths), **Greece** (46 deaths), **the Netherlands** (10 deaths), **Romania** (7 deaths), **Spain** (6 deaths), **France** (3 deaths), **Croatia** (1 death) and **Hungary** (1 death).

The number of human cases reported in the EU/EEA to date (1 715 cases) exceeds the average number reported during the same period over the past decade (693 cases), and is the highest number of WNV infections in humans ever reported in the EU/EEA.

As at 7 October 2026, locally acquired human cases have been reported in 187 NUTS 3 regions across 13 EU/EEA countries, making it the most geographically widespread WNV season on record.

As in previous years, most cases were reported among males aged 65 years and above. Overall, 77% of cases were hospitalised and 57% presented with neurological symptoms, compared with 10-year averages of 84% and 62%, respectively. The case fatality rate was approximately 8%, below the 10-year average of 10%. These findings are within expected levels and do not indicate increased disease severity. Country-specific case fatality rates are affected by the sensitivity of the surveillance system, including screening practices of blood donors, therefore direct comparisons between countries should be made with caution.

#### EU candidate countries

In 2026, and as at 7 October, the following EU candidate countries have reported 140 locally acquired human cases of WNV infection: **North Macedonia** (70 cases), **Serbia** (57 cases), **Kosovo*** (8 cases), **Albania** (4 cases) and **Moldova** (1 cases, of which 1 had an unknown place of infection). The earliest and latest dates of symptom onset among human cases were 12 May in North Macedonia and 28 September in Serbia, respectively. A total of 11 deaths have been reported by **North Macedonia** (10 deaths) and **Kosovo*** (1 death).

[1] Locally acquired cases refer to cases acquired within the reporting country

*This designation is without prejudice to positions on status and is in line with UNSCR 1244/1999 and the ICJ Opinion on the Kosovo declaration of independence.

### Findings from veterinary surveillance

#### EU/EEA countries

From the veterinary perspective, 13 countries in the EU/EEA have reported 510 WNV outbreaks among equids and 591 outbreaks among birds in 2026. The earliest reported WNV outbreak among equids had a suspected start date of 30 March 2026 in France, while the most recent had a suspected start date of 2 October 2026 in Germany. Outbreaks among equids have been reported by **France** (307 outbreaks), **Italy** (72 outbreaks), **Belgium** (45 outbreaks), **the Netherlands** (24 outbreaks), **Spain** (18 outbreaks), **Greece** (14 outbreaks), **Germany** (13 outbreaks), **Croatia** (5 outbreaks), **Austria** (4 outbreaks), **Portugal** (4 outbreaks), **Hungary** (3 outbreaks) and **Cyprus** (1 outbreak). Among birds, the earliest reported WNV outbreak had a suspected start date of 31 March 2026 in Italy, while the most recent had a suspected start date of 29 September 2026 in Austria. Outbreaks among birds have been reported by **Italy** (353 outbreaks), **Belgium** (166 outbreaks), **Germany** (18 outbreaks), **France** (14 outbreaks), **Croatia** (13 outbreaks), **Spain** (12 outbreaks), **Austria** (10 outbreaks), **Slovenia** (4 outbreaks) and **the Netherlands** (1 outbreak).

No information was available on the equid species involved in the outbreaks reported in ADIS. The bird species most frequently associated with the reported outbreaks were the hooded crow (138 outbreaks) and the common magpie (104), followed by the carrion crow (80), the Eurasian jackdaw (45), the Northern goshawk (27), common wood-pigeon (20), the Eurasian blackbird (19), the little owl (18), and the common kestrel (15). Several other bird species were also reported, each associated with up to 13 outbreaks.

As at 7 October 2026, the numbers of equid and bird outbreaks reported in July, August and September notably exceeded their respective historical monthly averages (2016–2025 for equids and 2022–2025 for birds), ranging from about two to more than five times the historical monthly averages for equids and remaining at about twice the corresponding averages for birds. The cumulative number of bird and equid outbreaks reported so far in 2026 is the highest recorded for the same period in previous years.

Overall, so far in 2026, outbreaks in birds and/or equids have been reported in 186 NUTS 3 regions across 13 EU/EEA countries. This exceeds the geographical spread observed during this period in 2025, when outbreaks were reported in 113 NUTS 3 regions across 11 countries, as well as what has been observed in most previous years. The 2026 season was instead comparable to 2024, which was one of the most geographically widespread WNV seasons for animal outbreaks, with outbreaks in birds and/or equids reported in 188 regions across 14 countries.

Of the 13 EU/EEA countries that submitted data to ADIS in 2026, all had previously reported WNV outbreaks in birds and/or equids, consistent with endemic WNV circulation.

#### EU candidate countries and other European countries

In 2026, as at 7 October, one EU candidate country (North Macedonia) and Switzerland had reported WNV outbreaks in birds and/or equids. **Switzerland** reported one outbreak in equids, with a suspected start date of 21 July 2026. **North Macedonia** reported four outbreaks in birds, with suspected start dates ranging from 18 August to 7 September 2026.

WNV outbreaks in animals were reported for the first time in the NUTS 3 region region of Aargau (CH033) and in two regions in North Macedonia. If outbreaks reported by both EU/EEA and other European countries are considered, the geographical spread observed to date in 2026 in Europe is slightly greater than what was observed during the corresponding period in 2024.

### Patterns across human and veterinary surveillance

#### EU/EEA countries

Most affected EU/EEA countries reported both human WNV infections and outbreaks in equids and/or birds. Romania only reported human WNV infections, while Portugal and Slovenia only reported outbreaks among equids and birds, respectively.

Differences in WNV patterns across European countries may reflect a combination of ecological, climatic and surveillance-related factors. Climatic conditions, habitat characteristics and the local abundance and distribution of competent mosquito vectors and bird hosts can influence WNV circulation. Differences in surveillance design, intensity, diagnostic practices and reporting may also affect the number of human infections and animal outbreaks detected.

Most human WNV infections are asymptomatic or subclinical. Consequently, the case numbers reported by EU/EEA countries represent only a fraction of the true number of infections. Furthermore, the reported numbers for of human cases and animal outbreaks, particularly for September, should be considered provisional, as reporting delays may lead to additional notifications in October or later.

A selection of country-specific findings is presented below to further illustrate the heterogeneity of WNV activity across Europe.

##### Italy

Italy remains the most affected country in 2026, reporting the highest number of WNV human cases (748, 44% of all reported human cases in the EU/EEA), including 56 deaths (case fatality rate: 7.5%). Italy also reported the highest number of outbreaks in birds (353; 59% of all avian outbreaks reported in the EU/EEA) and 72 outbreaks in equids (14% of all equid outbreaks reported in the EU/EEA). Nevertheless, the number of human cases reported was slightly lower than what was reported during the same period in 2025. As at 7 October 2026, the number of bird outbreaks notified to ADIS was the highest for the corresponding period since reporting became mandatory in 2021, while the number of equid outbreaks was higher than in the corresponding period of all previous years except 2018 and 2025. Overall, 79 of Italy’s 107 NUTS 3 regions are currently affected, with WNV human cases and/or animal outbreaks reported.

##### Greece

Greece has reported its highest number of human WNV cases for this time of year, with 445 cases, including 46 deaths (case fatality rate: 10%). WNV transmission is currently most intense in the Attica NUTS 2 region, which accounts for 218 cases, with the highest numbers reported in Anatoliki Attiki (East Attica, 102 cases) and Voreios Tomeas Athinon (North Athens, 63 cases). The Larisa NUTS 3 region accounts for 73 cases. Despite accounting for 26% of all reported human cases in the EU/EEA, Greece has reported only 14 outbreaks in equids, representing 2.7 % of all reported equid outbreaks, and no outbreaks in birds.

### France

France had reported 144 locally acquired human WNV infections, including three deaths (case fatality rate: 2%), accounting for 8.4% of all cases in the EU/EEA. This is by far the highest number of cases ever reported by France. France reported the highest number of equid outbreaks among EU/EEA countries, with 307 outbreaks, accounting for 52% of the 595 reported outbreaks. This was also the highest number of WNV outbreaks in equids ever notified by France. A further 45 outbreaks were reported in birds.

### The Netherlands

Fifty-nine human WNV infections have been reported in the Netherlands, including ten deaths (case fatality rate: 17%). The Netherlands previously reported locally acquired human WNV infections in 2020, when the country documented its first human cases, with seven cases reported in Utrecht (previously NL310) and one in the Arnhem/Nijmegen region (NL226). In addition, 24 outbreaks in equids and one in birds were reported in 2026. WNV was first reported in animals in the Netherlands in 2025, with a single equid outbreak. Overall, WNV infections in humans and/or animals were detected across 16 of the 40 NUTS 3 regions in the Netherlands this year.

### Belgium

Belgium has reported 15 human WNV infections, representing the country's first-ever reported human cases of WNV infection; no deaths were reported. Most cases had symptom onset in the second half of August. WNV was first detected in Belgium in 2025 through avian surveillance, with seven bird outbreaks reported in Arr. Mechelen (BE212), Arr. Dendermonde (BE232) and Arr. Halle-Vilvoorde (BE241). In 2026, WNV was again detected in birds in these three regions and spread to 15 additional NUTS 3 regions that had not previously reported WNV activity. Overall, 14 outbreaks in equids and 26 outbreaks in birds were reported across 18 of Belgium's 44 NUTS 3 regions in 2026.

### Newly affected regions

In 2026, the regions listed below reported WNV activity for the first time ever. The type of first reported event is indicated in brackets for each region, distinguishing between human WNV infections, animal outbreaks or both. WNV activity was reported for the first time ever in humans and/or animals by **Belgium** in Arr. Aalst (BE231, animal), Arr. Antwerpen (BE211, human, animal), Arr. de Bruxelles-Capitale/Arr. Brussel-Hoofdstad (BE100, human, animal), Arr. Charleroi (BE32B, animal), Arr. Eeklo (BE233, animal), Arr. Gent (BE234, animal), Arr. Hasselt (BE224, animal), Arr. Leuven (BE242, human, animal), Arr. Liège (BE332, animal), Arr. Maaseik (BE225, animal), Arr. Namur (BE352, animal), Arr. Nivelles (BE310, animal), Arr. Sint-Niklaas (BE236, human, animal), Arr. Tielt (BE257, animal) and Arr. Turnhout (BE213, animal); by **France** in Ain (FRK21, humans), Ariège (FRJ21, animal), Aude (FRJ11, human and animal), Bas-Rhin (FRF11, animal), Dordogne (FRI11, animal), Drôme (FRK23, human and animal), Essonne (FR104, animal), Haut-Rhin (FRF12, animal), Hauts-de-Seine (FR105, animal), Ille-et-Vilaine (FRH03, animal), Isère (FRK24, human), Landes (FRI13, animal), Loire-Atlantique (FRG01, human and animal), Lot-et-Garonne (FRI14, animal), Maine-et-Loire (FRG02, human and animal), Moselle (FRF33, animal), Pyrénées-Orientales (FRJ15, human and animal), Rhône (FRK26, human), Saône-et-Loire (FRC13, animal), Seine-et-Marne (FR102, human and animal), Tarn-et-Garonne (FRJ28, animal) and Val-d'Oise (FR108, animal); by **Germany** in Erzgebirgskreis (DED42, animal) and Heidelberg, Stadtkreis (DE125, animal); by **Italy** in Pescara (ITF13, human and animal), Rieti (ITI42, human and animal), Trento (ITH20, human and animal) and Valle d'Aosta/Vallée d'Aoste (ITC20, animal); by **the Netherlands** in Agglomeratie Leiden en Bollenstreek (NL363, human), Agglomeratie's-Gravenhage (NL361, human and animal), Delft en Westland (NL362, human animal), Groot-Amsterdam (NL32B, human and animal), Het Gooi en Vechtstreek (NL327, humans), IJmond (NL323, humans), Midden-Noord-Brabant (NL415, human), Noordoost-Noord-Brabant (NL416, human and animal), Oost-Zuid-Holland (NL365, human and animal), Veluwe (NL221, humans), Zuidoost-Zuid-Holland (NL364, human and animal) and Zuidwest-Gelderland (NL224, animal) and Zuidwest-Overijssel (NL212, humans) and by **Spain** in Girona (ES512, animal), Gran Canaria (ES705, animal) and Huesca (ES241, animal).

#### Other European countries

In 2026, WNV activity was reported for the first time in several regions of Albania, Kosovo, North Macedonia and Serbia: Korçë (AL034, human) in **Albania**; Ferizaj (XK005, human) and Gjakovë (XK007, human) in **Kosovo**; Pelagoniski (MK005, human and animal) and Vardarski (MK001, human) in **North Macedonia**; and Rasinska oblast (RS216, human) in **Serbia**.

It should be noted that other European countries, including EU candidate countries, have no obligation to notify animal outbreaks to ADIS.

##### North Macedonia

North Macedonia is reporting an unprecedented number of human WNV infections, with 70 cases and 10 deaths (case fatality rate: 14 %) reported from five NUTS 3 regions. Two of these regions have also reported outbreaks among birds. The Skopski region, which includes the capital city of Skopje, is by far the most affected, accounting for 61 human WNV infections and three bird outbreaks. In 2026, North Macedonia reported WNV outbreak data to ADIS for the first time, with four bird outbreaks across two regions. In the past decade, the country had reported a maximum of five human WNV infections per year.

### Seasonal outlook

In 2026, WNV activity peaked in humans at the end of August and in birds and equids in early September across Europe, followed by a marked decline.

Though environmental conditions for WNV transmission are becoming less favourable, low-level transmission may continue in the coming weeks and additional cases are still expected to be reported.

ECDC and EFSA continue to closely monitor the situation in Europe.

The most recent full automated report, including graphs, maps and a table, is available online at the following link:

https://www.ecdc.europa.eu/en/infectious-disease-topics/west-nile-virus-infection/surveillance-and-disease-data/monthly-updates

## Background

The tables and maps in this monthly report show the countries and areas where human cases of WNV infection and outbreaks among equids and birds have been reported to the European surveillance portal for infectious diseases (EpiPulse Cases) and the Animal Disease Information System (ADIS). In addition to this monthly report, ECDC provides a weekly report presenting the locally acquired WNV infections among humans in Europe[2]. Human surveillance covers EU/EEA countries as well as reporting by EU candidate and potential candidate countries. Cases are reported in accordance with the EU case definition for West Nile virus infection laid down in Commission Implementing Decision (EU) 2018/945. Some countries are already using (parts of) the updated case definition set out in Commission Implementing Regulation (EU) 2026/2097, which will formally apply from 1 July 2028 and replace the current definition.

For the analysis of ADIS outbreak data, this report includes only animal outbreaks with a 'suspicion/start date' in 2026. For analysis of historical data, when the 'suspicion/start date' was not reported, the 'confirmation date' was used instead. Outbreaks of WNV in animals shall be notified to ADIS in accordance with the provisions and definitions laid down in Commission Implementing Regulation (EU) 2020/2002. Under this Regulation, the reporting obligation applies to EU Member States. Data from non-EU countries, including candidate countries reporting through ADIS, may also be available in the system. As human surveillance does not cover Switzerland, animal outbreaks reported to ADIS by this country are presented exclusively in the 'Findings from veterinary surveillance' section. A notified outbreak may include one or more WNV cases. Since there is no specific case definition for WNV infection in animals, the general definition provided in Article 9 of Commission Delegated Regulation (EU) 2020/689 applies. When comparing the number of WNV outbreaks in birds reported in 2026 with historical data, a four-year average (2022–2025) was used – rather than the 10-year average applied to equids – since mandatory reporting of WNV outbreaks in birds only began in April 2021.

Surveillance of WNV in mosquitoes can support the detection of local virus circulation and complement human and animal surveillance findings. However, because these data are not systematically collected and reported at European level, they are not included in this report.

**EFSA staff consulted (in alphabetical order)**: Giusi Amore, Inma Aznar, Frank Verdonck

[2] In this report, Europe refers to EU/EEA countries, EU candidate countries (Albania, Bosnia and Herzegovina, Kosovo*, Moldova, Montenegro, North Macedonia, Serbia, Türkiye, and Ukraine) and Switzerland. Data from Switzerland are included only for animal outbreaks.
